# Paeonol Attenuates Cigarette Smoke-Induced Lung Inflammation by Inhibiting ROS-Sensitive Inflammatory Signaling

**DOI:** 10.1155/2014/651890

**Published:** 2014-08-03

**Authors:** Meng-Han Liu, An-Hsuan Lin, Hung-Fu Lee, Hsin-Kuo Ko, Tzong-Shyuan Lee, Yu Ru Kou

**Affiliations:** ^1^Department of Physiology, School of Medicine, National Yang-Ming University, Taipei 11221, Taiwan; ^2^Department of Neurosurgery, Cheng Hsin General Hospital, Taipei 11220, Taiwan; ^3^Department of Chest Medicine, Taipei Veterans General Hospital, Taipei 11217, Taiwan

## Abstract

Cigarette smoking causes persistent lung inflammation that is mainly regulated by redox-sensitive pathways. We have previously reported that cigarette smoke (CS) activates reactive oxygen species- (ROS-) sensitive mitogen-activated protein kinases (MAPKs)/nuclear factor-*κ*B (NF-*κ*B) signaling leading to induction of lung inflammation. Paeonol, the main phenolic compound present in the Chinese herb *Paeonia suffruticosa*, has antioxidant and anti-inflammatory properties. However, whether paeonol has similar beneficial effects against CS-induced lung inflammation remains unclear. Using a murine model, we showed that chronic CS exposure for 4 weeks caused pulmonary inflammatory infiltration, increased lung vascular permeability, elevated lung levels of chemokines, cytokines, and 4-hydroxynonenal (an oxidative stress biomarker), and induced lung inflammation; all of these CS-induced events were suppressed by chronic treatment with paeonol. Using human bronchial epithelial cells (HBECs), we demonstrated that cigarette smoke extract (CSE) sequentially increased extracellular and intracellular levels of ROS, activated the MAPKs/NF-*κ*B signaling, and induced interleukin-8 (IL-8); all these CSE-induced events were inhibited by paeonol pretreatment. Our findings suggest a novel role for paeonol in alleviating the oxidative stress and lung inflammation induced by chronic CS exposure *in vivo* and in suppressing CSE-induced IL-8 *in vitro* via its antioxidant function and an inhibition of the MAPKs/NF-*κ*B signaling.

## 1. Introduction

The inhalation of cigarette smoke (CS) causes chronic lung inflammation that leads to the development of chronic obstructive pulmonary disease (COPD) in smokers [1]. This CS-induced lung inflammation is well-recognized as being regulated by a complex mechanism involving various types of cells and inflammatory mediators [1, 2]. For example, chemokines and cytokines released from lung epithelial cells play a vital role in the regulation of lung inflammation because the lung epithelium is a target for direct insult by CS [3–8]. The induction of inflammatory mediators by CS in lung cells is mainly regulated by redox-sensitive signaling pathways [4, 6–9]. Initially, CS may increase the intracellular levels of reactive oxygen species (ROS) in lung epithelial cells [4, 7, 8] or other types of lung cells [9–12]. Subsequently, this increased intracellular ROS may activate various ROS-sensitive signaling pathways, such as the mitogen-activated protein kinases (MAPKs) and a number of downstream transcriptional factors, such as nuclear factor-*κ*B (NF-*κ*B), and ultimately promote inflammatory gene expression [4, 6–12]. The involvement of the ROS-sensitive signaling pathways suggests that therapeutic targeting of oxidative stress with antioxidants in order to improve lung inflammation should be beneficial when treating COPD [13].

Paeonol (2′-hydroxy-4′-methoxyacetophenone), the main phenolic compound of the radix of the Chinese herb* Paeonia suffruticosa* (*Cortex Moutan*), has been used as a traditional herbal medicine for thousands of years. Paeonol has been shown to suppress several inflammatory responses to stimuli other than CS in a number of animal models [14–18] and in various cell types [16, 19–23]. Furthermore, paeonol has been reported to possess antioxidant activity when used as* ex vitro* [24, 25],* in vitro* [21], and* in vivo* preparations [26]. Thus, the anti-inflammatory and antioxidant properties of paeonol make it a potential drug for the therapy of CS-induced lung inflammation. However, this possibility remains to be proven.

The aims of this study were, firstly, to investigate the antioxidant and anti-inflammatory effects of paeonol on CS-induced lung inflammation and, secondly, to determine the therapeutic mechanisms underlying the beneficial effects of paeonol. We employed an established murine model of chronic CS exposure [7, 8, 27] to assess the inhibitory effects of paeonol on oxidative stress and various indices of lung inflammation. Additionally, we used an established* in vitro* model of primary human bronchial epithelial cells (HBECs) [7, 8] to determine the suppressive effects of paeonol on increases in intracellular ROS, activation of the ROS-sensitive inflammatory signaling pathways, and the induction of interleukin-8 (IL-8), all of which are mediated by CS extract (CSE).

## 2. Materials and Methods

### 2.1. Reagents

Antibodies (Abs) and ELISA kits to measure IL-8, macrophage inflammatory protein 2 (MIP-2), monocyte chemoattractant protein-1 (MCP-1), keratinocyte chemoattractant (KC), and interleukin-1*β* (IL-1*β*) were purchased from R&D Systems (Minneapolis, MN, USA). Rabbit antibody against 4-hydroxynonenal (4-HNE) was purchased from Abcam (Cambridge, MA, USA). Antibodies against ERK, JNK, p65, and Histone H1 were obtained from Santa Cruz Biotechnology (Santa Cruz, CA, USA). Mouse antibody against *α*-tubulin, paeonol (purity > 99%, HPLC), N-acetyl-cysteine, and the 3-(4,5-dimethylthiazol-2-yl)-2,5 diphenyltetrazolium bromide (MTT) assay kit were purchased from Sigma-Aldrich (St. Louis, MO, USA). PD98059, SP600125, and BAY11-7085 were obtained from Calbiochem (San Diego, CA, USA). The membrane-permeable probe hydroethidine (HE) was purchased from Molecular Probes (Eugene, OR, USA).

### 2.2. Murine Model of Chronic CS Exposure and Paeonol Treatment

All animal experiments were approved by the Animal Care and Use Committee of the National Yang-Ming University. The murine model of chronic CS exposure has been described in detail previously [7, 8, 27]. Briefly, male C57BL/6J mice at the age of 8 weeks (National Laboratory Animal Center, Taipei, Taiwan) were randomly divided into four groups (*n* = 7 mice/group) for exposure to air or CS. These mice received daily treatment with paeonol (10 mg/kg) or saline (vehicle control) by gastric gavage during the 4-week exposure. The mice formed four groups, namely, Air, Air + paeonol, CS, and CS + paeonol. Animals were given* ad libitum* access to food and water, and the averaged body weights did not vary among the study groups after the 4-week exposure. For each CS exposure, the mice were placed in an exposure chamber and 750 mL of fresh CS generated from 1.5 cigarettes (Marlboro Red Label; 10.8 mg nicotine and 10.0 mg tar per cigarette) was delivered to the chamber. The CS passed out of the chamber via four exhaust holes (1 cm) on the side panels. During the exposure, the mice were conscious and breathed spontaneously in the chamber for 10 min. After exposure, the mice were transferred to a new cage and allowed to inspire air normally. The mice were exposed at 10:00 and 16:00 each day for 4 weeks. The control animals underwent identical procedures in another chamber but were only exposed to air. For each CS exposure, the particle concentration inside the exposure chamber was about 625 mg/m^3^ initially but decreased overtime due to the fact that the CS passed out of the chamber via the exhaust holes [8]. The HbCO levels immediately after the 10-minute exposure protocol for air-exposure and CS-exposure mice were 0.4% and 32%, respectively [8].

### 2.3. Preparation of Bronchoalveolar Lavage Fluid (BALF) and Lung Tissues

At the end of each experiment, the mice were euthanized with CO_2_ and a middle thoracotomy was performed. The left lung was ligated and the right lung was lavaged four times with 0.6 mL of warm PBS containing a complete protease inhibitor cocktail (Roche Diagnostics, Mannheim, Germany). The BALF samples were then centrifuged at 350 ×g for 5 min at 4°C, and the supernatant of the first lavage fluid was stored at −80°C for later analysis of total protein using a Bio-Rad protein assay reagent (Bio-Rad Laboratories, Inc., Hercules, CA, USA). The cell pellets of the BALF samples were resuspended in PBS for cell counting. Furthermore, the right lung was then stored at −80°C for subsequent analysis. The left lung was fixed with 4% paraformaldehyde and embedded in paraffin.

### 2.4. Histological Assessments

Formalin-fixed, paraffin-embedded tissue blocks were cut into 8 *μ*m sections. Sections were deparaffinized, rehydrated, and then underwent haematoxylin and eosin (H&E) staining and were viewed under a microscope (Motic TYPE 102M, Xiamen, China). The histological assessments were conducted by a pathologist who was blinded to the treatment. Each histological characteristic was scored on a scale of 0 (normal) to 5 (maximal). The lung inflammatory score was categorized according to the sum of the score for infiltration cell numbers and for damage level, including thickening of alveolar walls and epithelium, as well as increases in peribronchial and perivascular cuff area.

### 2.5. Determining the Concentrations of Chemokines and Cytokines

The concentrations of MIP-2, MCP-1, KC, and IL-1*β* in BALF and in lung tissue samples were measured using ELISA kits according to the manufacturer's instructions.

### 2.6. Measurement of an Oxidative Stress Biomarker

Level of 4-HNE modified proteins, a product of lipid peroxidation, in lung tissue samples was measured to serve as a biomarker of oxidative stress as described previously [28].

### 2.7. Preparation of CSE

CSE was freshly prepared on the day of the experiment as previously described [7, 8] with some modifications. In brief, 1000 mL of the smoke generated from two burning cigarettes (Marlboro Red Label, Philip Morris, Richmond, VA, USA) without filters was sucked under a constant flow rate (8 mL/s) into a syringe and then bubbled into a tube containing 20 mL serum-free medium. The CSE solution was sterilized using a 0.22 *μ*m filter (Millipore, Bedford, MA, USA) and the pH was adjusted to 7.4. The optical density of the CSE solution was determined by measuring the absorbance at 302 nm [29] or 320 nm [30] which in reality showed little difference between different preparations. This CSE solution was considered 100% CSE and was further diluted with serum-free medium to various desired concentrations that were then used to treat HBECs for different duration times.

### 2.8. Cell Culture

HBECs (Cascade Biologics, Portland, OR, USA) were cultured in epithelial cell growth medium (Medium 200; Cascade Biologics) containing 10% fetal bovine serum (FBS), 1x low serum growth supplement, 100 U/mL penicillin, 100 *μ*g/mL streptomycin, and 0.25 *μ*g/mL amphotericin B (Biological Industries, Kibbutz Beit Haemek, Israel) at 37°C in an incubator with 5% CO_2_.

### 2.9. Cell Viability Assay

Cell viability was measured by the MTT assay as described previously [31]. Briefly, cells were incubated with or without paeonol for 24 hours and 100 *µ*L of MTT (0.5 mg/mL in medium) was then added. The cells incubated with control medium were considered 100% viable.

### 2.10. Measurement of Extracellular and Intracellular ROS Levels

The membrane-permeable probe HE was used to assess levels of ROS using methods that have been described previously [31]. Oxidation of HE by ROS forms red fluorescent ethidium (ETH) [32]. HBECs were incubated in culture medium containing 10 *μ*M HE at 37°C for 30 min. After stimulation with CSE for the desired time, the culture medium was removed for the measurement of extracellular ROS levels. The cells were then washed and detached with trypsin/EDTA to allow measurement of intracellular ROS levels. Since our cells were preincubated with the ROS-sensitive probe, the amount of ROS generated in the medium or cells during the duration of the CSE challenge could be reflected by the fluorescent intensities. The fluorescence intensities of the culture medium and cells were analyzed using a multilabel counter (PerkinElmer, Waltham, MA, USA) at 530 nm excitation and 620 nm emission for ETH. Images of the cells were also obtained by examining them using a Nikon TE2000-U florescence microscope (Tokyo, Japan).

### 2.11. Western Blot Analysis

The cell lysates were prepared using cell lysis buffer (Cell Signaling, Beverly, MA, USA). Nuclear extracts were prepared by a method that has been reported previously [33] with modifications. Aliquots of cell lysates, nuclear extracts, or tissue lysates were separated by 8–12% SDS-PAGE and then transblotted onto Immobilon-P membrane (Millipore, Billerica, MA, USA). After being blocked with 5% skim milk, the blots were incubated with various primary antibodies and then the appropriate secondary antibodies. The specific protein bands were detected using an enhanced chemiluminescence kit (PerkinElmer), which was followed by the quantification with the ImageQuant 5.2 software (Healthcare Bio-Sciences, Philadelphia, PA, USA).

### 2.12. Statistical Analysis

The results are presented as mean ± SEM. Statistical evaluations involved one-way ANOVA followed by Dunnett's test or Fisher's least significant difference procedure for multiple comparisons were used as appropriate. Differences were considered statistically significant at *P* < 0.05.

## 3. Results

### 3.1. Effect of Paeonol on Inflammatory Manifestations in Mice

**Figure 1:**
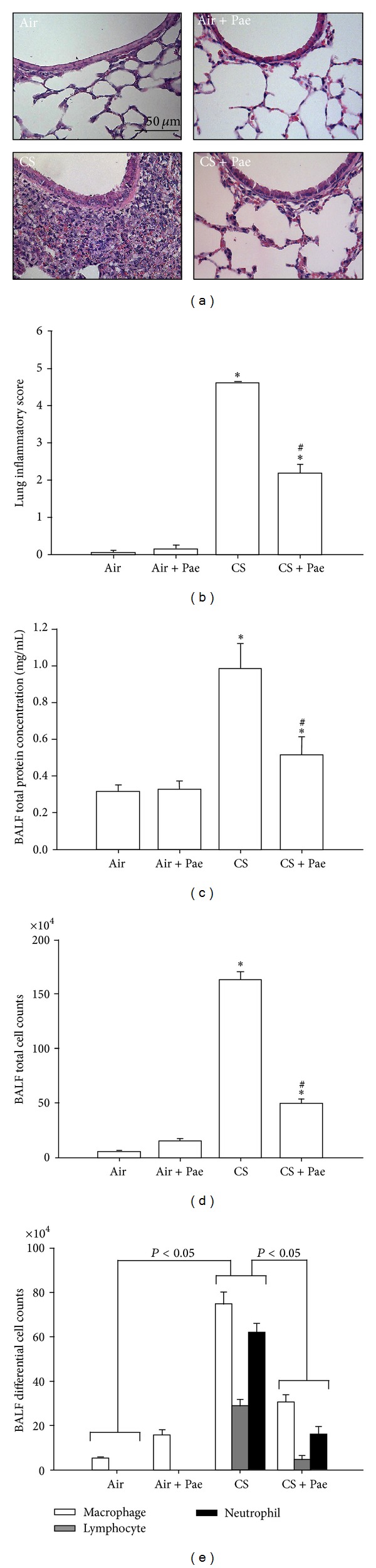
Paeonol (Pae) attenuates cigarette smoke- (CS-) induced lung inflammation in mice. Four groups of mice were chronically exposed to air or CS for 4 weeks. Two of the four study groups received daily treatment with paeonol (10 mg/kg body weight) or saline (vehicle control) by gastric gavage during the 4-week exposure. (a) Representative images of H&E stained lung sections. (b) Lung inflammatory scores were calculated according to the sum of the levels of cell infiltration and damage levels as assessed from the lung sections. ((c)–(e)) Total protein content, total cell count, and differential cell count in bronchoalveolar lavage fluid (BALF) were measured and served as indications of lung inflammation. Data in each group are mean ± SEM from 7 mice. *P < 0.05 versus the air-exposure group; ^#^ *P* < 0.05 versus the CS-exposure group with vehicle treatment.

Exposure of mice to CS for four weeks resulted in the development of lung inflammation. A histological evaluation of the H&E stained lung sections (Figure 1(a)) revealed extensive infiltration of inflammatory cells, thickening of the alveolar walls, and the presence of abnormal reepithelialization in the CS-exposure mice, all of which were found to be less in the CS-exposure mice that underwent paeonol treatment. The difference in the degree of histopathological manifestations between the CS-exposure mice with and without paeonol treatment was confirmed by comparisons of the group data in terms of lung inflammatory scores (Figure 1(b)). Furthermore, relative to the air-exposure mice, the CS-exposure mice were found to show increases in total protein levels (Figure 1(c)), total cell counts (Figure 1(d)), and differential cell counts (Figure 1(e)) in BALF. All of these inflammatory indices were significantly alleviated in the CS-exposure mice that underwent paeonol treatment (Figure 1). These inflammatory manifestations were not found in the air-exposure mice that underwent paeonol treatment (Figure 1).

### 3.2. Effect of Paeonol on Increases in Chemokines and Cytokines in Mice

**Figure 2:**
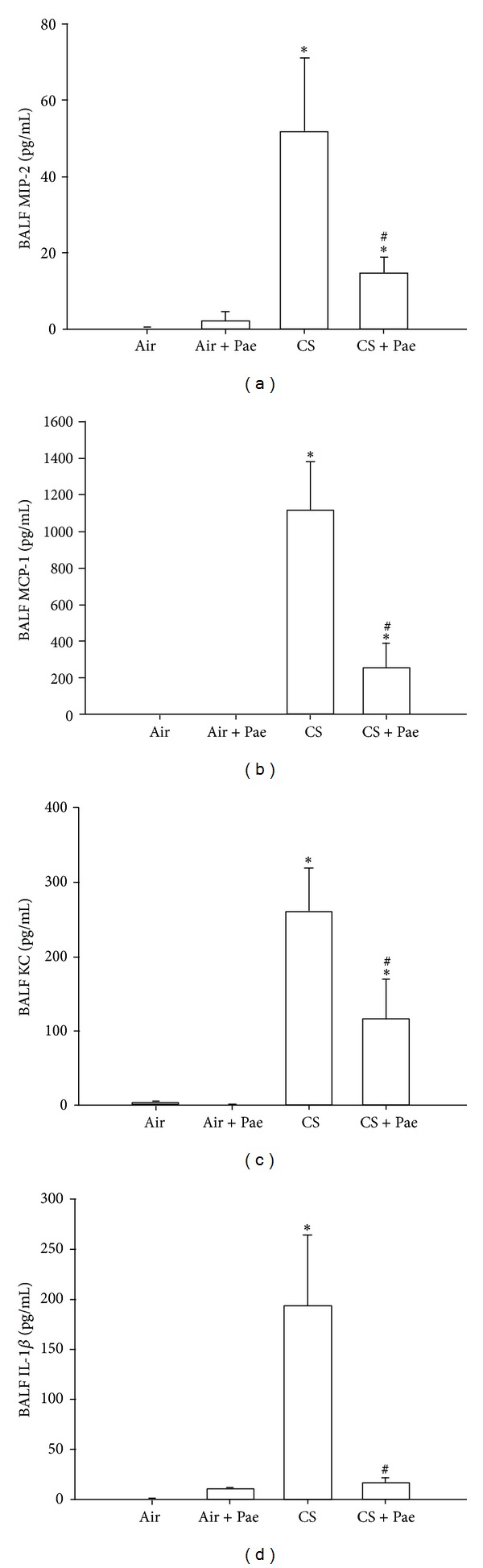
Paeonol (Pae) suppresses cigarette smoke- (CS-) induced increases in proinflammatory chemokines and cytokines in bronchoalveolar lavage fluid (BALF) sampled from mice. Levels of macrophage inflammatory protein 2 (MIP-2; (a)), monocyte chemoattractant protein-1 (MCP-1; (b)), keratinocyte chemoattractant (KC; (c)), and interleukin-1*β* (IL-1*β*; (d)) in BALF were analyzed by ELISA. Data in each group are mean ± SEM from 7 mice. *P < 0.05 versus the air-exposure group; ^#^ *P* < 0.05 versus the CS-exposure group with vehicle treatment. See legend of Figure 1 for detailed information on each study group.

**Figure 3:**
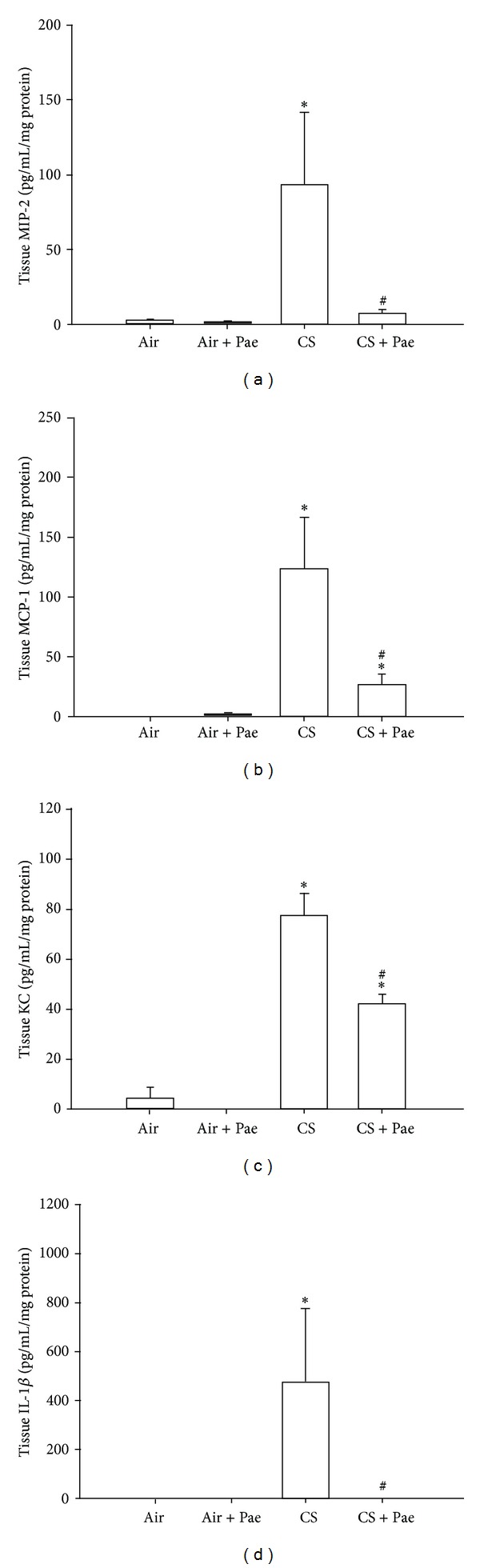
Paeonol (Pae) suppresses cigarette smoke- (CS-) induced increases in proinflammatory chemokines and cytokines in lung tissues sampled from mice. Levels of macrophage inflammatory protein 2 (MIP-2; (a)), monocyte chemoattractant protein-1 (MCP-1; (b)), keratinocyte chemoattractant (KC; (c)), and interleukin-1*β* (IL-1*β*; (d)) in lung tissues were analyzed by ELISA. Data in each group are mean ± SEM from 7 mice. *P < 0.05 versus the air-exposure group; ^#^ *P* < 0.05 versus the CS-exposure group with vehicle treatment. See legend of Figure 1 for detailed information on each study group.

Various chemokines and cytokines are important for the development of CS-induced lung inflammation [1, 2]. Relative to the air-exposure mice, exposure of mice to CS resulted in increases in the levels of MIP-2, MCP-1, KC, and IL-1*β* in BALF (Figure 2) and lung tissue samples (Figure 3). These increases in the levels of these chemokines and cytokines were found to be greatly reduced in the CS-exposure mice that underwent paeonol treatment (Figures 2 and 3). Paeonol treatment did not produce significant changes in levels of these chemokines and cytokines in the air-exposure mice (Figures 2 and 3).

### 3.3. Effect of Paeonol on Increases in Expression of Inflammatory Target Proteins and on the Presence of Oxidative Stress in Mice

**Figure 4:**
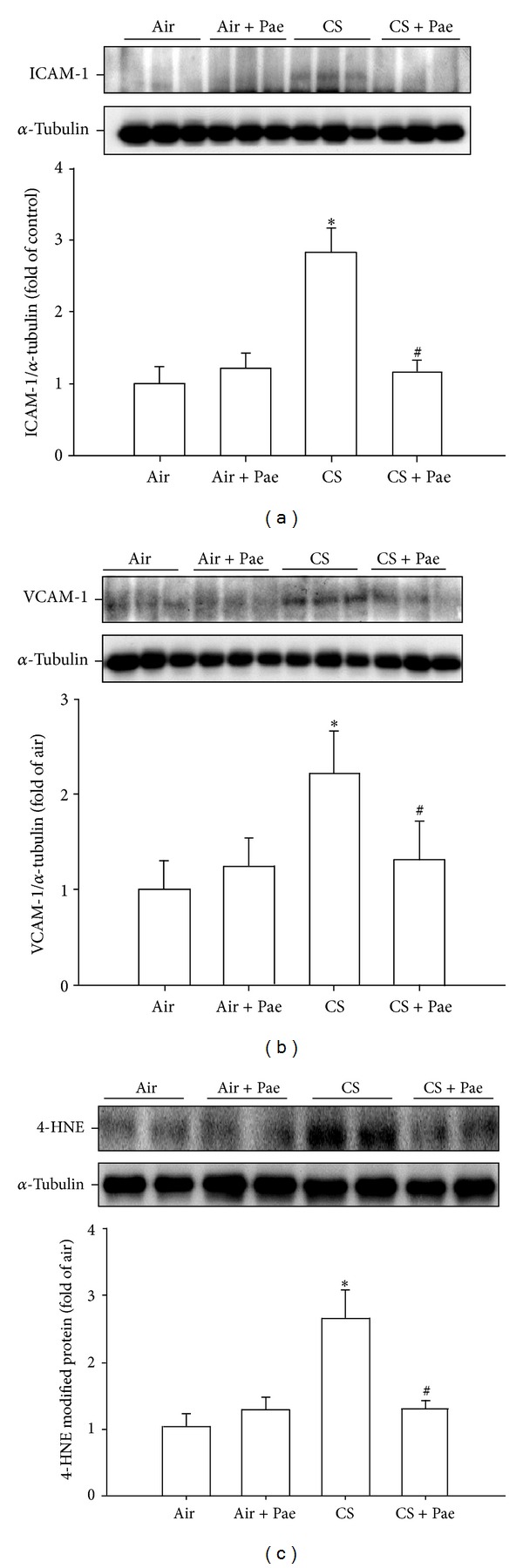
Paeonol (Pae) suppresses cigarette smoke- (CS-) induced increases in expression of inflammatory target proteins ((a), (b)) and oxidative stress-related proteins (c) in lung tissues samples from mice. (a) Intercellular adhesion molecule 1 (ICAM-1); (b) vascular cell adhesion molecule 1 (VCAM-1); (c) 4-hydroxynonenal (4-HNE) modified proteins (a biomarker of oxidative stress). Protein levels in the lung tissues were analyzed by Western blotting. Data in each group are mean ± SEM from 7 mice. *P < 0.05 versus the air-exposure group; ^#^ *P* < 0.05 versus the CS-exposure group with vehicle treatment. See legend of Figure 1 for detailed information on each study group.

ICAM-1 and VCAM-1 are inflammatory target proteins [10], whereas 4-HNE is a biomarker of oxidative stress [28]. Relative to the air-exposure mice, exposure of mice to CS resulted in increases in expression of ICAM-1 (Figure 4(a)) and VCAM-1 (Figure 4(b)) as well as an increase in the presence of 4-HNE modified proteins (Figure 4(c)) in lung tissues. These increases in the expression of ICAM-1 and VCAM-1 and the presence of 4-HNE modified proteins were found to be significantly attenuated in the CS-exposure mice that underwent paeonol treatment (Figure 4). Paeonol treatment did not produce significant changes in the expression of ICAM-1 and VCAM-1 and presence of 4-HNE modified proteins in the air-exposure mice (Figure 4).

### 3.4. Effect of Paeonol on the Induction of IL-8 in HBECs

**Figure 5:**
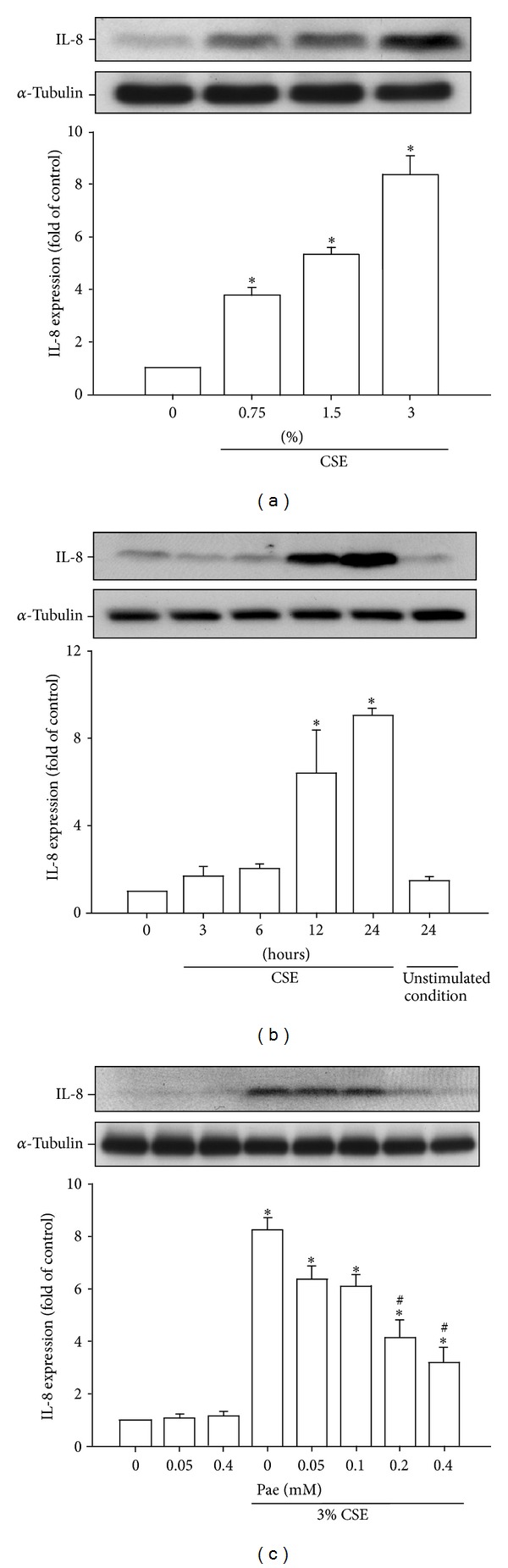
Paeonol (Pae) dose-dependently attenuates the induction of IL-8 by cigarette smoke extract (CSE) in human bronchial epithelial cells. (a) Cells were exposed to 0–3% CSE for 24 hours. (b) Cells were incubated with medium alone (control) or 3% CSE for indicated times. (c) Cells were incubated with medium alone or 3% CSE for 24 hours with pretreatment with various concentrations (0–0.4 mM) of Pae. Protein levels of IL-8 in the cell lysates were analyzed by Western blotting. Data in each group are mean ± SEM from four independent experiments. *P < 0.05 versus control ((a), (c)) or zero time (b). ^#^ *P* < 0.05 versus CSE without Pae pretreatment (c).

We additionally used HBECs as an* in vitro* model to study the therapeutic mechanism of paeonol. Exposure of HBECs to various concentrations (0, 0.75, 1.5, and 3%) of CSE for 24 hours concentration-dependently increased the protein level of IL-8 (Figure 5(a)). In addition, exposure of HBECs to 3% CSE for up to 24 hours time-dependently increased the level of IL-8 protein (Figure 5(b)). The use of 3% CSE was therefore chosen as the standard treatment for all subsequent experiments throughout this study. Pretreatment with paeonol dose-dependently attenuated the induction of IL-8 by CSE, whereas pretreatment with paeonol in cells without CSE stimulation failed to alter the expression of IL-8 (Figure 5(c)). Results obtained from MTT assay indicated that exposure of HBECs to paeonol (0.4 mM) for 24 hours did not alter the cell viability (100.8 ± 4.3% of control).

### 3.5. Effect of Paeonol on Increases in Extracellular and Intracellular ROS in HBECs

**Figure 6:**
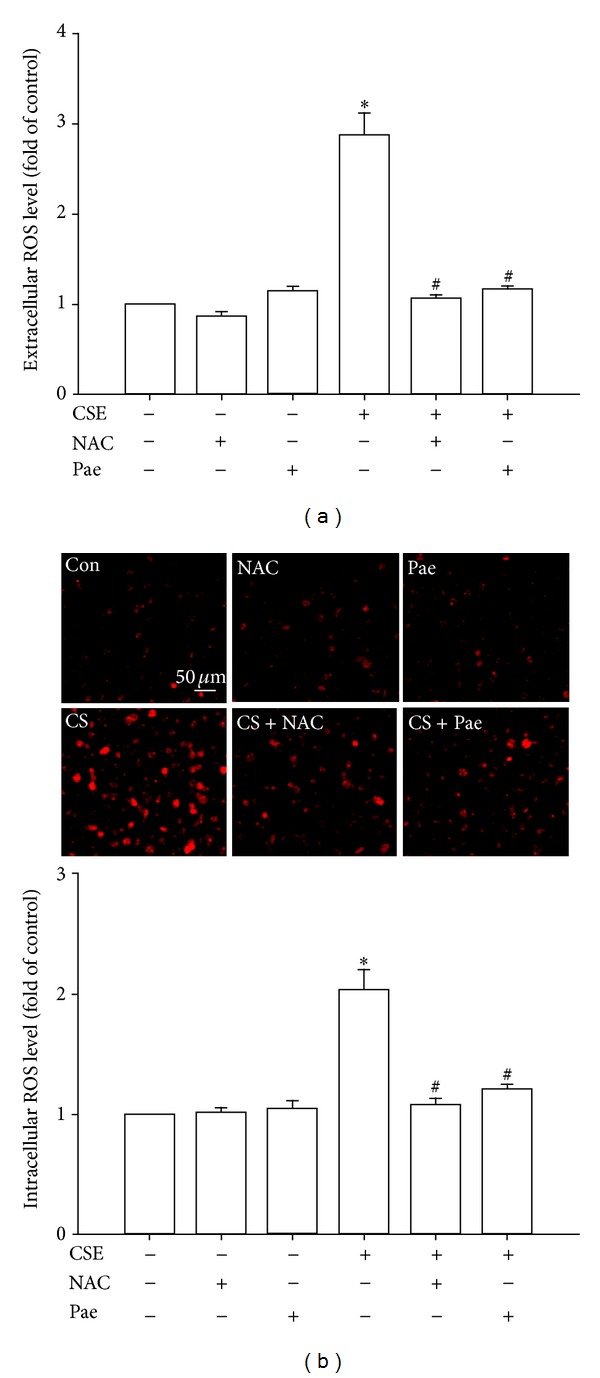
Paeonol (Pae) attenuates increases in extracellular and intracellular levels of reactive oxygen species (ROS) induced by cigarette smoke extract (CSE) in human bronchial epithelial cells. Cells were exposed to medium alone or 3% CSE for 2 (a) and 30 minutes (b) with pretreatment with N-acetyl-cysteine (NAC, 2 mM; a ROS scavenger), Pae (0.4 mM), or the vehicle of Pae. After exposure, the culture medium was removed for the measurement of extracellular ROS levels (a). The cells were collected for the measurement of intracellular ROS levels (b). Levels of ROS were measured by HE/ETH fluorescent probe assay. Data in each group are mean ± SEM from four independent experiments. *P < 0.05 versus control. ^#^ *P* < 0.05 versus CSE without drug pretreatment.

ROS are an important trigger for the induction of IL-8 by CSE in HBECs [4, 7, 8]. Relative to control cells, exposure of HBECs to 3% CSE for 2 and 30 minutes resulted in increases in both the extracellular (Figure 6(a)) and intracellular levels (Figure 6(b)) of ROS, respectively. Both the CSE-induced increases in extracellular and intracellular ROS were prevented by pretreatment with either paeonol or N-acetyl-cysteine (a ROS scavenger that served as a positive control) (Figure 6). Pretreatment with paeonol or N-acetyl-cysteine in cells without CSE stimulation failed to alter the levels of ROS (Figure 6).

### 3.6. Effect of Paeonol on the Activation of MAPKs/NF-*κ*B Signaling

**Figure 7:**
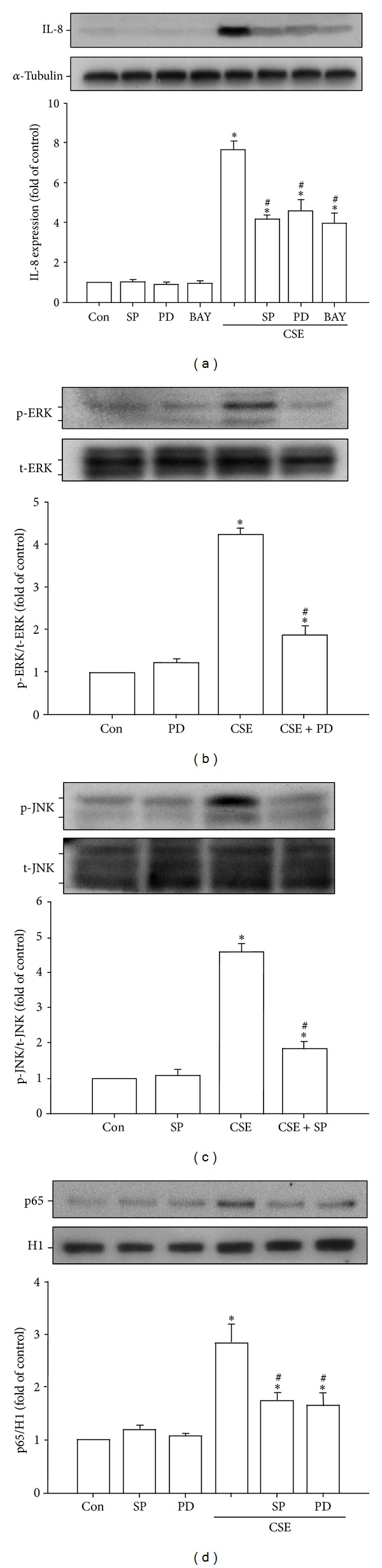
The ERK, JNK, and NF-*κ*B signaling is crucial for the induction of IL-8 by cigarette smoke extract (CSE) in human bronchial epithelial cells. Cells were exposed to medium alone or 3% CSE for 24 (a), 6 ((b), (c)), or 12 hours (d) with pretreatment with an ERK inhibitor (PD98059; PD, 10 *μ*M), a JNK inhibitor (SP600125; SP, 10 *μ*M), or a NF-*κ*B inhibitor (BAY 11-7085; BAY, 10 *μ*M). Protein expression was analyzed by Western blotting. Activation of ERK (b) or JNK (c) was indicated by increased phosphorylation of these kinases in cell lysates, whereas activation of NF-*κ*B (d) was indicated by increased presence of p65 subunit in the cell nucleus. *P < 0.05 versus control (Con). ^#^ *P* < 0.05 versus CSE without inhibitor pretreatment.

**Figure 8:**
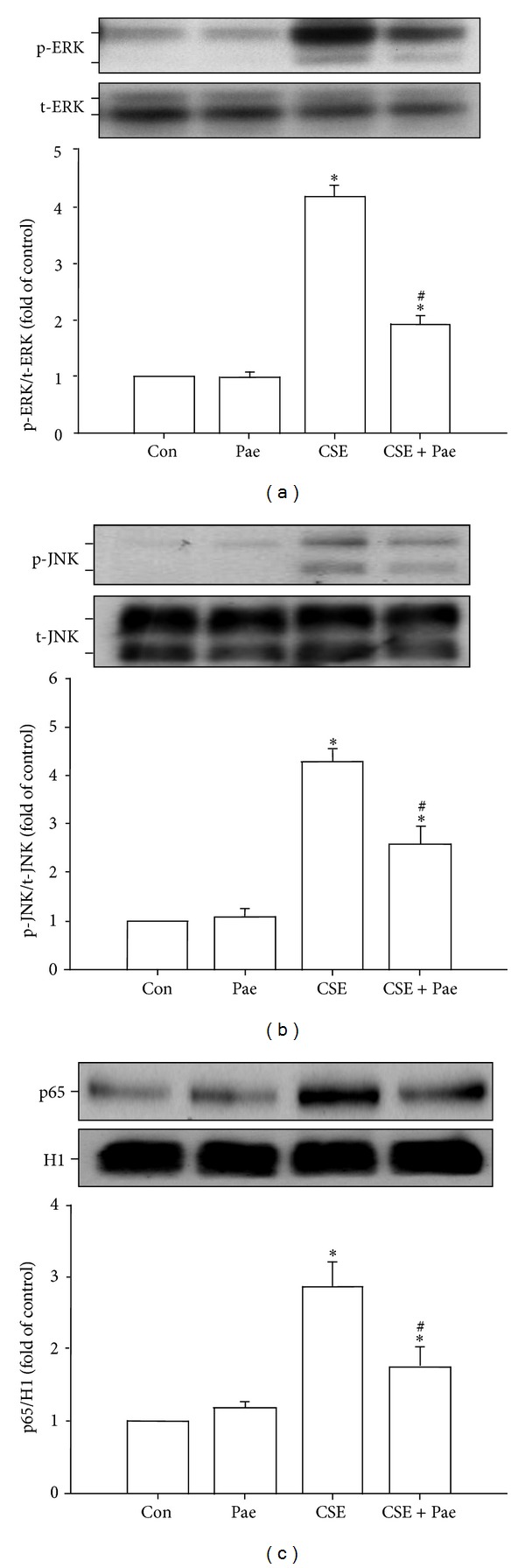
Paeonol (Pae) attenuates the activation of ERK, JNK, and NF-*κ*B signaling by cigarette smoke extract (CSE) in human bronchial epithelial cells. Cells were exposed to medium alone or 3% CSE for 6 ((a), (b)) or 12 hours (c) with pretreatment with Pae (0.4 mM) or the vehicle of Pae. Protein expression was analyzed by Western blotting. *P < 0.05 versus control. ^#^ *P* < 0.05 versus CSE without Pae pretreatment.

The activations of ERK, JNK, and NF-*κ*B are known to be a crucial ROS-sensitive signaling pathway that is central to the induction of IL-8 by CSE in HBECs [4, 6–8]. We found that pretreatment of HBECs with an ERK inhibitor (PD98059), a JNK inhibitor (SP600125), or a NF-*κ*B inhibitor (BAY 11-7085) was able to significantly reduce CSE-mediated IL-8 expression (Figure 7(a)). Additionally, relative to control cells, exposure of HBECs to 3% CSE for 6 hours resulted in increases in the amount of phosphorylated ERK (Figure 7(b)) and phosphorylated JNK (Figure 7(c)), both of which were suppressed by pretreatment with their inhibitors. Furthermore, exposure of HBECs to 3% CSE for 12 hours resulted in an increase in the amount of NF-*κ*B p65 subunit present in the nuclei of cells, which was reduced by pretreatment with either the ERK inhibitor or JNK inhibitor (Figure 7(d)). Such CSE-induced activation of the MAPKs/NF-*κ*B signaling was significantly attenuated by pretreatment with paeonol (Figure 8). Pretreatment with paeonol in cells without CSE stimulation failed to alter the expression of these proteins (Figure 8).

## 4. Discussion

Our* in vitro* study demonstrates that chronic CS exposure of mice for 4 weeks caused lung inflammation as evidenced by histopathological manifestations, several inflammatory indices (BALF cell counts and protein levels), and increases in presence of inflammatory chemokines and cytokines (MIP-2, MCP-1, KC, and IL-1*β*) in BALF and lung tissue samples. Chronic CS exposure also caused increases in expression of inflammatory target proteins (ICAM-1 and VCAM-1) and in oxidative stress (4-HNE) in mice. All of these CS-induced events are similar to those described previously [2, 3, 7, 8]. Importantly, all of these CS-induced events were suppressed by chronic treatment with paeonol, suggesting that paeonol has anti-inflammatory and antioxidant functions against CS-induced lung inflammation* in vivo*. We then used an* in vitro* model to investigate the therapeutic mechanism underlying the beneficial effects of paeonol. We employed HBECs to study the induction of IL-8 by CSE because IL-8 produced by lung epithelial cells is known to be important to the induction of lung inflammation by CS [3–8]. We demonstrate that exposure of HBECs to CSE sequentially increased both extracellular and intracellular ROS levels, activated both MAPKs and NF-*κ*B, and induced IL-8. Previous studies investigating the upstream and downstream relationships among these events have revealed the importance of the ROS-sensitive MAPKs/NF-*κ*B signaling pathway to the induction of IL-8 by CSE in lung epithelial cells [4, 6–8], which was confirmed by our results obtained from the experiments using pharmacological inhibitors for ERK, JNK, and NF-*κ*B. Importantly, all of these CSE-induced consequences were suppressed by pretreatment with paeonol, indicating that the beneficial effect of paeonol may be mediated through its antioxidant activity and the inhibition of the ROS-sensitive inflammatory signaling.

Our study appears to be the first to report that paeonol has both antioxidant and anti-inflammatory activity against CS-induced lung inflammation. These two beneficial activities of paeonol have been suggested by previous studies that have focused on stimuli other than CS insult. Using animal models, paeonol has been shown to suppress the inflammation in lipopolysaccharide- (LPS-) induced lung injury [14, 15], ovalbumin-induced hyperresponsive airways [16], atherosclerosis [17], carrageenan-evoked thermal hyperalgesia [18], and ischemia-reperfusion brain injury [26]. Using* in vitro* preparation, paeonol has been demonstrated to inhibit the inflammatory responses to LPS in macrophages [16, 19, 20], in microglial cells [20, 21], in fibroblasts [22], and in TNF-*α*-stimulated endothelial cells [23]. Particularly, the anti-inflammatory effect of paeonol* in vitro* appears to be due to inactivation of the signaling pathways involved [14, 19–21, 23]. In addition to its anti-inflammatory activity, paeonol has been reported to scavenge ROS* ex vitro* [24, 25]. Paeonol also possesses antioxidant activity against oxidative stress in cortical neurons* in vitro* [21] and in ischemia-reperfusion brain injury* in vivo* [26]. Thus, our findings are in good agreement with the above reported observations.

In our* in vitro* study, the increases in extracellular (within 2 minutes) and intracellular ROS (within 30 minutes) are two very early events after CSE exposure. Indeed, CS is a potent oxidant that can directly generate ROS in extracellular fluid [7, 34] and in BALF, which is the lining fluid of the lung epithelium [7, 35]. Certain types of extracellular ROS, such as H_2_O_2_, may diffuse through the cell membrane, contributing to the increase in intracellular ROS [36]. Additionally, CS may also increase intracellular ROS via activation of NADPH oxidase in lung epithelial cells [7, 8] or other types of lung cells [11, 12, 37, 38] shortly after exposure. Whatever the sources of ROS, paeonol was able to effectively prevent increases in both extracellular and intracellular ROS, suggesting it may serve as a ROS scavenger, as proposed by other investigators [24–26]. Alternatively, the antioxidant function of paeonol has been suggested to be mediated through the prevention of activation of NADPH oxidase and upregulation of the antioxidant system in microglial cells [21]; while the former mechanism may be possible, the latter is unlikely because the time (30 min) perhaps is too short for the upregulation of an antioxidant system. In the present study, we also demonstrate that the MAPKs/NF-*κ*B signaling is crucial for the induction of IL-8 by CSE. Thus, once the CSE-induced intracellular ROS were removed by paeonol, it is reasonable to observe the CSE-induced activation of the MAPKs/NF-*κ*B signaling was lessened because this pathway is ROS-sensitive [4, 6–8]. However, we cannot exclude the possibility that paeonol directly interferes with the activation of this signaling pathway because this has been suggested in LPS-treated macrophages [14, 19, 20] and in microglial cells [20, 21], as well as in TNF-*α*-activated endothelial cells [23].

Our* in vivo* studies demonstrate that the paeonol-mediated attenuation of CS-induced lung inflammation is associated with a reduction of lung oxidative stress, which is consistent with our* in vitro* findings. The level of 4-HNE modified protein reflects the degree of lipid peroxidation by total ROS in lung tissues [28], which encompass the ROS generated from CS and the ROS released from infiltrated inflammatory cells such as macrophages and neutrophils [1]. Since the infiltration of these inflammatory cells into the lung was alleviated in paeonol-treated mice, it is reasonable to observe a reduced lung oxidative stress in these mice. In addition, it is still unknown whether the beneficial effects of paeonol are limited to the induction of chemokines and cytokines in lung epithelial cells or also apply to other cell types such as leukocytes. If the latter is true, the target cells for paeonol would not be confined to the lung epithelial cells because the paeonol was given systemically to the mice in this study.

In our* in vitro* study, the dose of paeonol did not produce any notable cytotoxicity as revealed by the assay for cell viability. In our* in vivo* study, the dose used is only one tenth of the dose used in previous studies in mice [16, 17] and was chosen to avoid possible adverse effects. Paeonol has a minimal systemic toxicity (LD_50_ = 3430 mg/kg) when orally administered to mice [39]. Since the dose used in this study is far less than the LD_50_ dose, we did not measure parameters of blood chemistry and did not evaluate the toxicity of the drug to liver and kidney. The Chinese herb* Paeonia suffruticosa* has been shown to possess therapeutic effects such as sedation, hypnosis, antipyresis, analgesic, anti-inflammation, and immunoregulation [39]. Although paeonol has been extensively employed to investigate its beneficial effects in several animal models of diseases, its clinical use as a single compound is limited. For this reason, future application of paeonol in the clinical settings should consider its potential side effects and possible herb-drug interactions. The other limitation of this study is that we measured the levels of MIP-2, MCP-1, KC, and IL-1*β* in the* in vivo* study but only assessed the production of IL-8 in the* in vitro* study. MIP-2, MCP-1, and KC are potent chemokines and IL-1*β* is an important cytokine for the initiation and progression of lung inflammation induced by cigarette smoke [40]. MIP-2 and KC are the murine IL-8 homologues and, therefore, we decided to investigate the production of IL-8 in the* in vitro* study.

In summary, our findings suggest a novel role for paeonol regarding the alleviation of oxidative stress and lung inflammation induced by chronic CS exposure* in vivo*, and the suppression of the CSE-induced IL-8* in vitro* by inhibiting MAPKs/NF-*κ*B signaling, possibly via its antioxidant function. Our findings support the possibility of using paeonol to ameliorate lung inflammation in smokers and that paeonol treatment may be a potential therapy option when treating COPD. Future research should attempt to investigate the therapeutic potential of the ingredients of other herbs or dietary supplements as antioxidants for treatments of CS-induced lung inflammation.
